# The use of insecticide treated nets by age: implications for universal coverage in Africa

**DOI:** 10.1186/1471-2458-9-369

**Published:** 2009-10-01

**Authors:** Abdisalan M Noor, Viola C Kirui, Simon J Brooker, Robert W Snow

**Affiliations:** 1 Malaria Public Health and Epidemiology Group, Centre for Geographic Medicine, KEMRI - University of Oxford - Wellcome Trust Collaborative Programme, Kenyatta National Hospital Grounds (behind NASCOP), P.O. Box 43640-00100, Nairobi, Kenya; 2 Centre for Tropical Medicine, Nuffield Department of Clinical Medicine, University of Oxford, CCVTM, Oxford OX3 7LJ, UK; 3 Department of Infectious and Tropical Diseases, London School of Hygiene and Tropical Medicine, London, UK

## Abstract

**Background:**

The scaling of malaria control to achieve universal coverage requires a better understanding of the population sub-groups that are least protected and provide barriers to interrupted transmission. Here we examine the age pattern of use of insecticide treated nets (ITNs) in Africa in relation to biological vulnerabilities and the implications for future prospects for universal coverage.

**Methods:**

Recent national household survey data for 18 malaria endemic countries in Africa were assembled to indentify information on use of ITNs by age and sex. Age-structured medium variant projected population estimates for the mid-point year of the earliest and most recent national surveys were derived to compute the population by age protected by ITNs.

**Results:**

All surveys were undertaken between 2005 and 2009, either as demographic health surveys (n = 12) or malaria indicator surveys (n = 6). Countries were categorized into three ITN use groups: <10%; 10 to <20%; and ≥20% and projected population estimates for the mid-point year of 2007 were computed. In general, the pattern of overall ITNs use with age was similar by country and across the three country groups with ITNs use initially high among children <5 years of age, sharply declining among the population aged 5-19 years, before rising again across the ages 20-44 years and finally decreasing gradually in older ages. For all groups of countries, the highest proportion of the population not protected by ITNs (38% - 42%) was among those aged 5-19 years.

**Conclusion:**

In malaria-endemic Africa, school-aged children are the least protected with ITNs but represent the greatest reservoir of infections. With increasing school enrollment rates, school-delivery of ITNs should be considered as an approach to reach universal ITNs coverage and improve the likelihood of impacting upon parasite transmission.

## Background

The slow progress toward the target set by African heads of state in Abuja in 2001 of 60% coverage of insecticide-treated nets (ITNs) among vulnerable children and pregnant women [1] has, in recent years, shown promising signs of changing with rapid scaling-up of ITNs coverage in many African countries [2]. However the Abuja target, and the Millennium Develop Goal 6 (80% coverage of children and pregnant women) [3], do not account for scaling ITN to achieve high coverage of all population segments necessary to reduce malaria transmission and protect communities. The scaling of ITNs demands a shift from prioritizing vulnerable populations to protecting everyone, including the most vulnerable, by achieving high coverage and community-wide use of ITNs. ITNs at high coverage levels impact vector population survival and abundance, where those not sleeping under an ITN will benefit and a mass-effect is achieved. The latter has been observed during trials of ITNs during the 1990's [4-8]. Theoretical models strongly support the likely benefit of levels of coverage beyond those most vulnerable to the clinical burden posed by *Plasmodium falciparum *[9,10]. Underpinning these models is the fact that it is estimated that 80% of human-to-mosquito transmission originates from human hosts older than 5 years of age, with *P. falciparum *prevalence, under stable malaria transmission, rising during early childhood, peaking in older children and falling through adolescence and adulthood [11].

There are now extensive temporal data on ITNs coverage across Africa since 2000 generated as part of national household cluster sample surveys [2,12,13]. These data have been used to examine progress toward coverage of ITNs among children under five years of age and pregnant women [2,12,14] or determinants of use [15,16]. Inevitably survey tools and indicators were developed around international targets established 10 years ago and thus most data focus on coverage of ITNs among the vulnerable groups or provide some indication of ownership among households. Notable is the paucity of data presented on coverage and use by age and sex across the entire surveyed community.

Following recent calls for universal coverage of ITNs and other vector control strategies [17], and given the biological basis for the target, we have analyzed datasets from those recent national surveys that describe coverage by age and sex among all members of a household.

## Methods

Household survey data on coverage of ITNs (defined as pretreated nets obtained within the last 12 months or nets that have been soaked with insecticide within the past 12 months or a long lasting insecticidal net (LLIN)) were sought from three principal sources: a) demographic and health surveys (DHS) [18]; b) malaria indicator surveys (MIS) undertaken by national malaria programmes using a package of standardized tools developed by the Roll Back Malaria [19]; and c) multiple indicators cluster surveys (MICS) supported by the United Nations Children's Fund [20]. These surveys are designed to be nationally representative with a sample size often of more than 3,000 households derived from a two-stage cluster sample design and are typically conducted every 3-5 years in collaboration with national ministries of health and statistics bureaus. Not all surveys contained information on ITNs use among all household members. No national surveys undertaken before 2005 contained information on ITNs use by all ages. 32 malaria endemic countries had undertaken national surveys since 2005 where data were available for download (14 DHS; 6 MIS; and 12 MICS). None of the MICS reported ITNs use by all ages. MIS have been completed for Mozambique (2007), Ethiopia (2007) and Eritrea (2008) but these data were not available online at the time of analysis. For Ethiopia, however, the DHS 2005 which contained ITNs use by all ages was used instead. In Congo and Liberia, DHS were completed between 2005 and 2007 but neither had information on ITNs use among all household members. Information was therefore available for a detailed analysis of the age and sex patterns of ITNs use among all household members from 18 malaria endemic countries in Africa undertaken between 2005 and 2009 as part of DHS (n = 12) or MIS (6) (Table 1). Plots of ITNs use overall and by gender were constructed by age for each country separately (see Additional file 1). Countries were ranked based on the proportion of individuals of all ages sleeping under an ITNs the night before survey and were then aggregated into three groups - Country Group 1 (≥ 20%: Kenya, Tanzania, Mali, Zambia), Country Group 2 (10% - < 20%: Benin, Senegal, Angola, Djibouti, Sudan) and Country Group 3 (<10%: Rwanda, Uganda, Namibia, Niger, DRC, Zimbabwe, Ethiopia, Guinea, Swaziland). To estimate the numbers of individuals unprotected in each country the medium variant age-structured projected population estimates for 2007 from the United Nation's World Population Prospects database was used [21]. The period 2007 was selected as the mid-point of the assembled national surveys all of which were undertaken between 2005 and 2009.

**Table 1:** Summary of national households surveys with data on ITN use among all age-groups in African countries where national surveys reported information on all ages: countries are ranked from highest to lowest based on the proportion of individuals of all ages who slept under an ITN the night prior to survey.

	**Country**	**Survey**	**Year**	**Month**	**Number of clusters sampled for survey**	**Number of households samples for survey**	**Number of persons seen during survey**	**Number of ITN owned by the sampled households**	**% of household with at least two ITN**	**% of children <5 years old sleeping under ITN the night before survey**	**% of persons of all ages sleeping under ITN the night before survey**
**Group 1**	Kenya	MIS	2007	June-July	200	6,854	31,297	5,483	22.5	39.2	38.9
	Tanzania	AIS/MIS	2007-8	October-February	475	8,497	41,871	5,948	18.8	32.3	29.4
	Zambia	DHS	2007	April-October	319	7,164	35,562	8,486	24.9	29.1	23.9
	Mali	DHS	2006	April-September	408	12,998	73,685	15,621	25.2	27.3	21.2

**Group 2**	Benin	DHS	2006	July-November	750	17,511	90,650	12,790	10.3	19.7	14.1
	Senegal	MIS	2006	November-December	150	3,063	30,199	3,998	21.7	16.2	13.2
	Angola	MIS	2006-7	November-April	120	2,599	14,399	1,040	10.9	17.7	13.0
	Djibouti	MIS	2008-9	December-February	156	3,603	22,373	1,802	17.6	19.9	13.0
	Sudan	MIS	2005	October	143	2,460	10,639	1,194	4.8	15.4	11.3

**Group 3**	Rwanda	DHS	2005	February-July	462	10,272	47,851	4,498	4.2	13.6	9.4
	Uganda	DHS	2006	May-October	368	8,870	45,439	3,291	5.8	9.6	7.2
	Namibia	DHS	2006-7	October-March	500	9,200	42,633	2,562	8	11.1	6.0
	Niger	DHS	2006	January-June	345	7,660	47,964	2,633	12.8	8.7	5.5
	DRC	DHS	2007	May-August	300	8,886	48,291	2,567	1.9	7.7	5.3
	Zimbabwe	DHS	2005-6	August-March	400	9,285	40,805	929	2.5	3.0	3.3
	Ethiopia	DHS	2005	April-August	540	13,721	67,539	589	0.3	2.3	1.5
	Guinea	DHS	2005	February-June	297	6,282	38,182	409	0.2	1.4	1.1
	Swaziland	DHS	2006-7	July-February	275	4,843	22,143	86	1.2	0.7	0.4

AIS = AIDS Indicator Survey; DRC = Democratic Republic of Congo; ITN = Insecticide Treated Net (a bed net treated with an insecticide the last six months prior to survey or a long lasting insecticidal net); DHS = Demographic and Health Survey; MIS = Malaria Indicator Survey.

## Results

**Figure 1:**
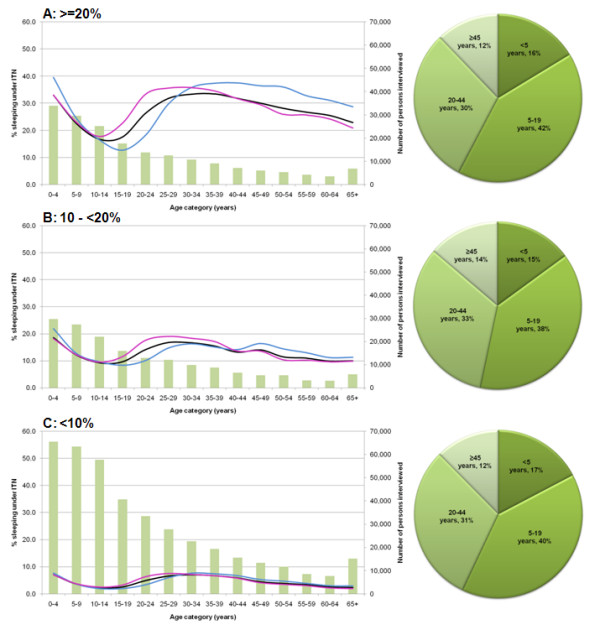
**Graphs of percentage of sample population sleeping under ITN the night before survey, overall and by gender, against the number of persons enumerated in each age category (left) and pie charts of the proportion of projected 2007 population who did not sleep under ITN by age category (right) in: A) group 1: countries where ≥ 20% of the sample population slept under ITN (Kenya, Tanzania, Zambia, Mali); B) group 2: countries where 10-<20% of the sample population slept under ITN (Benin, Senegal, Angola, Djibouti, Sudan); C) group 3 - countries where<10% of the sample population slept under ITN (Rwanda, Uganda, Namibia, Niger, DRC, Zimbabwe, Ethiopia, Guinea, Swaziland)**. Pink, blue and black lines on the graphs represent the percentage female, male and total sample population sleeping under ITN the night before survey respectively.

In general, the pattern of overall ITNs use with age was similar by country (see Additional file 1) and across the three country groups with ITNs use initially high among children <5 years of age, sharply declining among the population aged 5-19 years, before rising again across the ages 20-44 years and finally decreasing gradually in older ages (Table 1, Figures 1a-1c). This trend, however, was more pronounced with increasing overall ITNs coverage (Figure 1a). When the pattern of ITNs use was viewed by sex, the data showed that in the age group <5 years, a higher proportion of male children slept under ITNs compared to female in Country Groups 1 and 2 but no difference in Country Group 3. Between the ages of 10-34 years in Country Groups 1 and 3 and 10-44 years in Country Group 2 more females slept under ITNs compared to the males.

Population estimates and ITNs use information were arranged into four age categories: <5 years; 5-19 years; 20-44 years; and ≥45 years, corresponding to the traditional ITNs target groups of young children; older children and adolescents; adults most likely to have young children; and older household members at the tip of the population pyramid respectively. The proportion of individuals protected with ITNs and the total number of people not protected in each of the four age groups were recomputed and used to estimate the numbers of people un-protected in each country (Table 2). For all groups of countries, the highest proportion of the population not protected by ITNs (38% - 42%) was among those aged 5-19 years (Table 2 & Figures 1a-c).

**Table 2:** A summary of ITN use among individuals of ages < 5 years; 5-19 years; 20-44 years; and ≥45 years and the estimated number of persons (millions) in each age group NOT protected with ITN in 2007: countries are ranked from highest to lowest based on the proportion of individuals of all ages who slept under an ITN the night prior to survey.

**Country**	**Millions children < 5 years in 2007 (% sleeping under ITN)**	**Millions children < 5 years of age NOT sleeping under ITN in 2007**	**Millions children 5-19 years in 2007 (% sleeping under ITN)**	**Millions children 5-19 years of age NOT sleeping under ITN in 2007**	**Millions of persons 20-44 years in 2007 (% sleeping under ITN)**	**Millions of persons 20-44 years of age NOT sleeping under ITN in 2007**	**Millions of person ≥ 45 years of age in 2007 (% not sleeping under ITN)**	**Millions of persons ≥ 45 years of age NOT sleeping under ITN in 2007**
Kenya	6.32 (39.2)	3.80	14.14 (30.0)	9.90	12.91 (43.5)	7.29	4.46 (35.5)	2.87
Tanzania	7.34 (32.3)	4.97	15.61(21.5)	12.25	13.25 (32.5)	8.94	5.22 (26.2)	3.82
Zambia	2.23 (29.1)	1.58	4.81 (16.4)	4.02	3.83 (29.6)	2.70	1.48 (26.8)	1.08
Mali	2.15 (27.3)	1.56	4.77 (15.1)	4.05	4.07 (24.8)	3.06	1.43 (23.3)	1.10
Benin	1.41 (19.7)	1.13	3.12 (10.6)	2.79	2.74 (17.2)	2.27	1.13 (10.4)	1.01
Senegal	1.99 (16.2)	1.67	4.59 (11.3)	4.07	3.97 (13.5)	3.43	1.37 (14.4)	1.17
Angola	3.14 (17.7)	2.58	6.81 (7.5)	6.30	5.55 (15.7)	4.68	2.07 (13.6)	1.79
Djibouti	0.11 (19.9)	0.09	0.30 (11.8)	0.26	0.30 (12.7)	0.27	0.13 (10.3)	0.11
Sudan	5.80 (15.4)	4.90	14.69 (9.2)	13.34	13.95 (11.5)	12.34	6.06 (9.5)	5.48
Rwanda	1.61 (13.6)	1.39	3.56 (5.0)	3.38	3.21 (13.8)	2.77	1.13 (6.9)	1.05
Uganda	6.00 (9.6)	5.42	12.52 (4.4)	11.97	9.18 (10.4)	8.23	3.04 (6.4)	2.85
Namibia	0.27 (11.1)	0.24	0.76 (4.3)	0.72	0.75 (6.8)	0.70	0.31 (4)	0.30
Niger	2.97 (8.7)	2.71	5.52 (4.2)	5.28	4.11 (6.2)	3.85	1.63 (3.7)	1.57
DRC	11.64 (7.7)	10.75	24.69 (3.2)	23.90	18.92 (6.9)	17.61	7.33 (4.4)	7.01
Zimbabwe	1.71 (3.0)	1.66	5.01 (1.7)	4.92	4.23 (4.7)	4.03	1.60 (3.0)	1.55
Ethiopia	13.08 (2.3)	12.78	30.37 (1)	30.07	24.82 (1.9)	24.35	10.52 (0.9)	10.42
Guinea	1.61 (1.4)	1.59	3.58 (0.7)	3.56	3.11 (1.5)	3.06	1.36 (0.9)	1.35
Swaziland	0.16 (0.7)	0.16	0.46 (0.1)	0.46	0.38 (0.7)	0.38	0.15 (0.1)	0.15

## Discussion

Among the 18 national, household surveys analyzed across a range of overall ITNs coverage settings a common pattern of reported ITNs use emerges with highest coverage among children aged less than five years, dropping to lowest levels of coverage among children and adolescents aged 5-19 years and rising again through adulthood before a drop among the oldest household members (Figures 1a-1c). Similar differentials of ITNs use between young children and older age groups have been reported during studies in Tanzania [22-24], South Central Somalia [25], Ethiopia [26] and Nigeria [27]. The two most plausible and linked explanations for these observed patterns are that first most ITNs delivery programmes have historically focused on ensuring young children have access to nets either through routine clinic visits, attendance at regular vaccination visits, their mothers while pregnant or nets delivered as part of mass-catch-up immunization campaigns that target young children [28]. Consequently this age group would be expected, following recent efforts to scale coverage, to show the highest reported ITNs use. However, secondly this will be linked to the way people share sleeping structures in a household, where nursing and younger children will sleep with their mothers and/or both parents, who will most often be between 20-44 years of age. Conversely older children will sleep on separate beds or mats elsewhere in the household.

**Figure 2:**
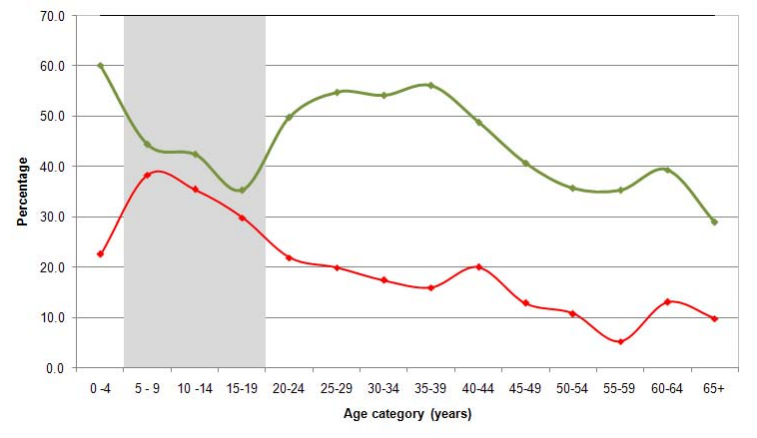
**Graph showing the prevalence of infection (red line) among individuals of all ages in the Coast province of Kenya prior to scaled ITN delivery **[31]**and the proportion of the population sleeping under an insecticide treated net (green line) in 2007 after the national free mass campaign of 2006 **[32]. The graph illustrates that in Kenya the national ITNs scaling-up strategies have been sub-optimal in terms of targeting the populations aged 5-19 years (shaded grey), the age-group in which pre-intervention parasite prevalence was at its peak.

If scaled delivery of ITNs to young children continues to increase it may be possible to reach a point of saturation within a household as nets are shared among older siblings and other household members. However this would depend largely on the longevity of the intact net and the insecticide. A more likely scenario is that redundancy will emerge for ITNs acquired by younger children within five years and these will be disposed of by the household or require replacement by national malaria control programmes. The group most neglected by current ITNs delivery strategies is children and adolescents aged between 5-19 years. This is a particularly important group for two reasons: a) they represent a large fraction of the population in most developing African communities (green bars shown in Figures 1a-1c); and b) while they may have developed a functional immune response against clinical disease before their fifth birthday [29,30] they have not developed a parasitic immunity that regulates the risk of blood stage infection. To highlight this vulnerability, Figure 2 shows age-stratified estimates of ITNs use from the 2007 Kenya MIS for populations living at the coast and pre-intervention infection prevalence among populations living in the same area [31]. As with national estimates, ITNs coverage in Coastal Kenya [32] is lowest among those aged 5-19 years, a period when the prevalence of infection reaches a peak and represents the sustained highest levels of infection prevalence in the community. Notably, ITNs coverage increased in all segments of the population, but in the 5-19 year old age group, the increase in ITNs coverage was smallest of any age group. As expected this group represents the largest threat to the success of scaled, universal coverage of ITNs likely to impact upon reduced community-levels of transmission.

Operations research in Africa show that the pre-existing infrastructure of schools can cost-effectively deliver simple health interventions, including deworming and micronutrients, as well as feeding programmes [33]. In areas of high enrollment, where the majority of non-enrolled school age children have at least one sibling attending school and few differentials in enrollment by socio-economic and health status exists [34], school health programmes are likely to be extremely equitable. Even in areas of low enrollment, non-enrolled children can still benefit from school health programmes: experience in several African countries demonstrates that many out-of-school children will take advantage of services, such as deworming, provided through schools [33]. Such features of school-based programmes provide a potentially equitable and cost-effective framework for malaria control [34]. Already, drug-based approaches to the prevention of malaria infection and anaemia in this target population are being considered again [35-38] after popular chemoprophylaxis strategies for school children in Africa during the 1950s and 1960's [39-41]. Given the poor coverage of current ITN programmes as a means to prevent infection among school aged children, pragmatic trials or operational investigations of the impact of ITNs delivered to children attending school should be compared to the provision, separately or in combination, with drugs used for intermittent presumptive treatment. In addition there is need to increase effective communication to households to encourage optimum usage of ITNs to address the widespread problem of households often using only a proportion of the nets they own while some household members remain unprotected [42]. Effective use of these reserve nets will also reduce redundancy in ITNs distributions by national programmes.

## Conclusion

In conclusion, the study shows that in malaria endemic African countries, school-age children are the least protected with ITNs. School-delivery of ITNs, therefore, should be considered as an approach to reach universal coverage and improve the likelihood of impacting upon malaria parasite transmission. As most sub-Saharan African countries move towards universal coverage of ITNs it becomes important that national survey data can be used to redefine optimal approaches to this new strategy. Therefore data on ITN use must be collected for all household members and not, as is the case with the MICs surveys and some DHS surveys, for those only under the age of five years and pregnant women.

## Abbreviations

DHS: Demographic and Health Surveys; MIS: Malaria Indicator Surveys; MICS: Multiple Indicators Cluster Surveys; ITN: Insecticide Treated Nets; MDG: Millennium Development Goals.

## Competing interests

The authors declare that they have no competing interests.

## Authors' contributions

AMN was responsible for data cleaning, analysis, interpretation and production of the final manuscript. VCK downloaded all the survey data, reconstructed the data and conducted preliminary analysis. SJB provided advice on analysis, interpretation of results and helped with the preparation of final manuscript. RWS was responsible for overall scientific management, analysis, interpretation and preparation of the final manuscript. All authors have read and approved the final version of the manuscript.

## Pre-publication history

The pre-publication history for this paper can be accessed here:



## Supplementary Material

Additional file 1 **Use of insecticide treated nets by all ages**. Plots of use of insecticide treated nets by age for each of the 18 countries presented in the manuscript Click here for file
