# Supplementary material for: The use of insecticide treated nets by age: implications for universal coverage in Africa
Source: BMC Public Health. 2009 Oct 1;9:369. doi: 10.1186/1471-2458-9-369 (PMC2761895; doi:10.1186/1471-2458-9-369)
Supplement: Additional file 1 — Use of insecticide treated nets by all ages. Plots of use of insecticide treated nets by age for each of the 18 countries presented in the manuscript [file 1471-2458-9-369-S1.DOC]

**Additional File 1: Use of insecticide treated nets by all ages**

Pink line represents the percentage female sleeping under an ITN the night before survey; blue line represents the percentage male sleeping under an ITN the night before survey; black line represents the percentage of all persons sleeping under an ITN the night before survey; and the green bars show the number of individuals who were interviewed during survey.

**Group 1 (≥20% ITN use among all ages)**

**Kenya (MIS 2007)**


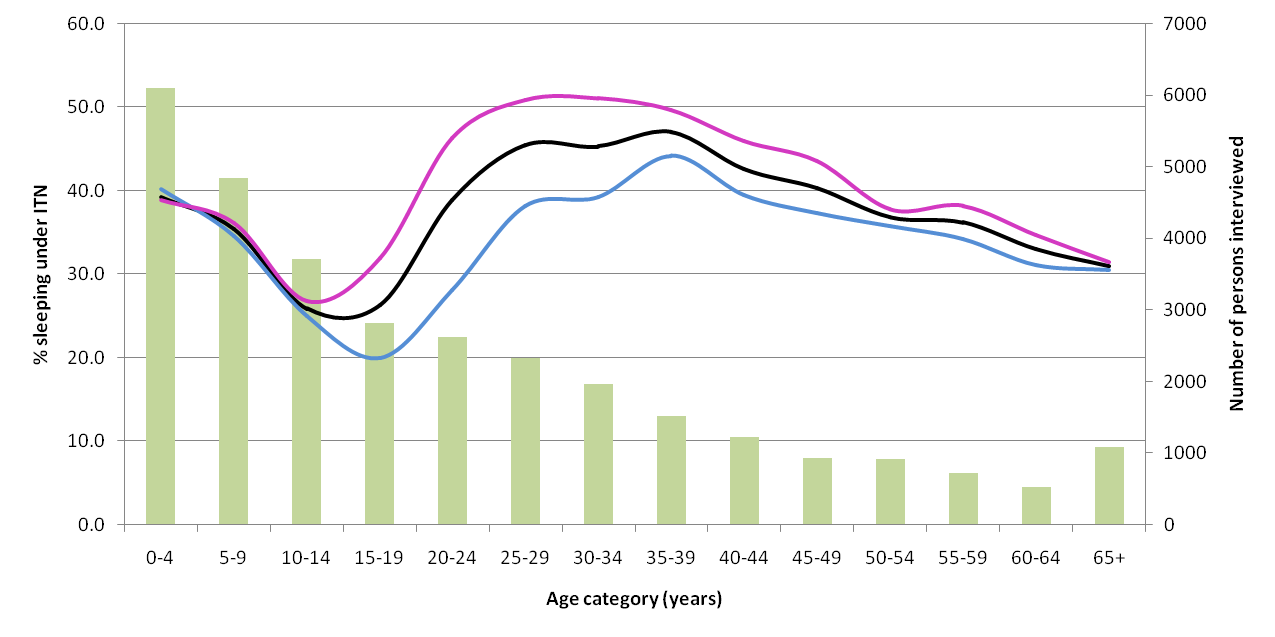


**Tanzania (ASI-MIS 2007-8)**


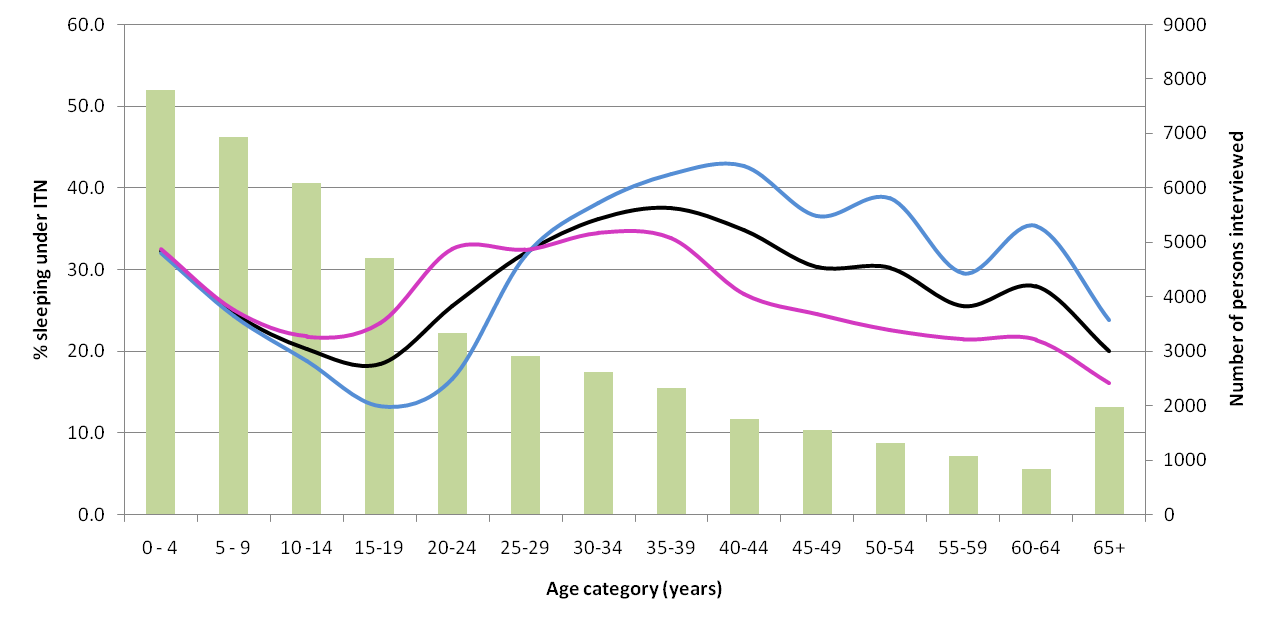


**Zambia (DHS 2007)**


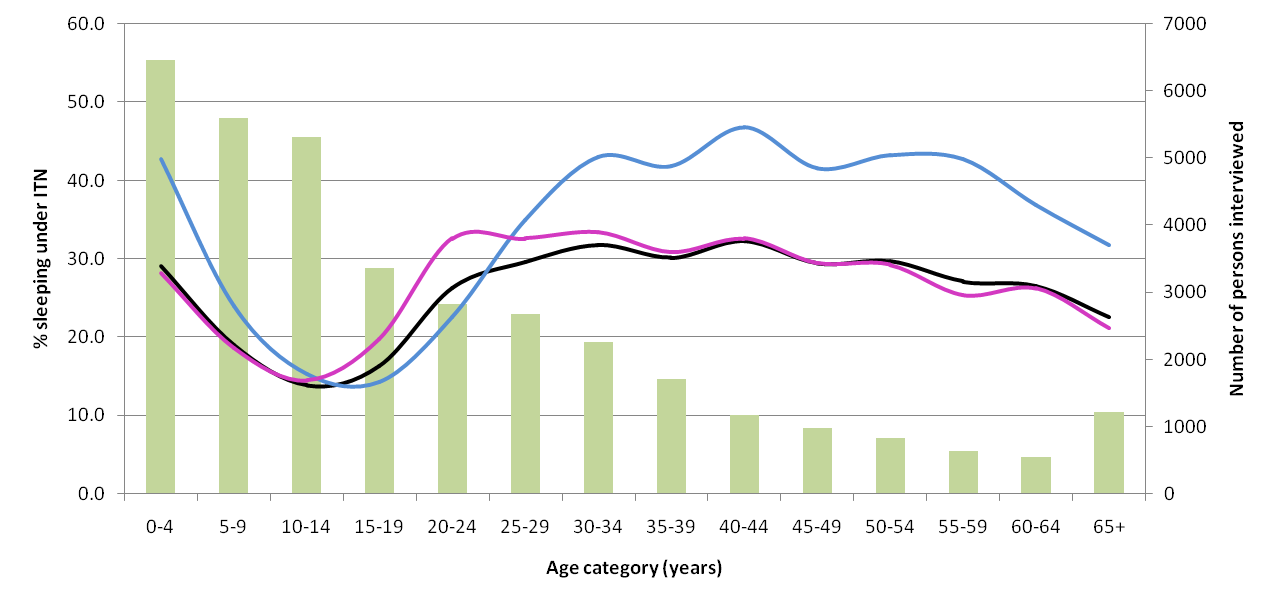


**Mali (DHS 2006)**


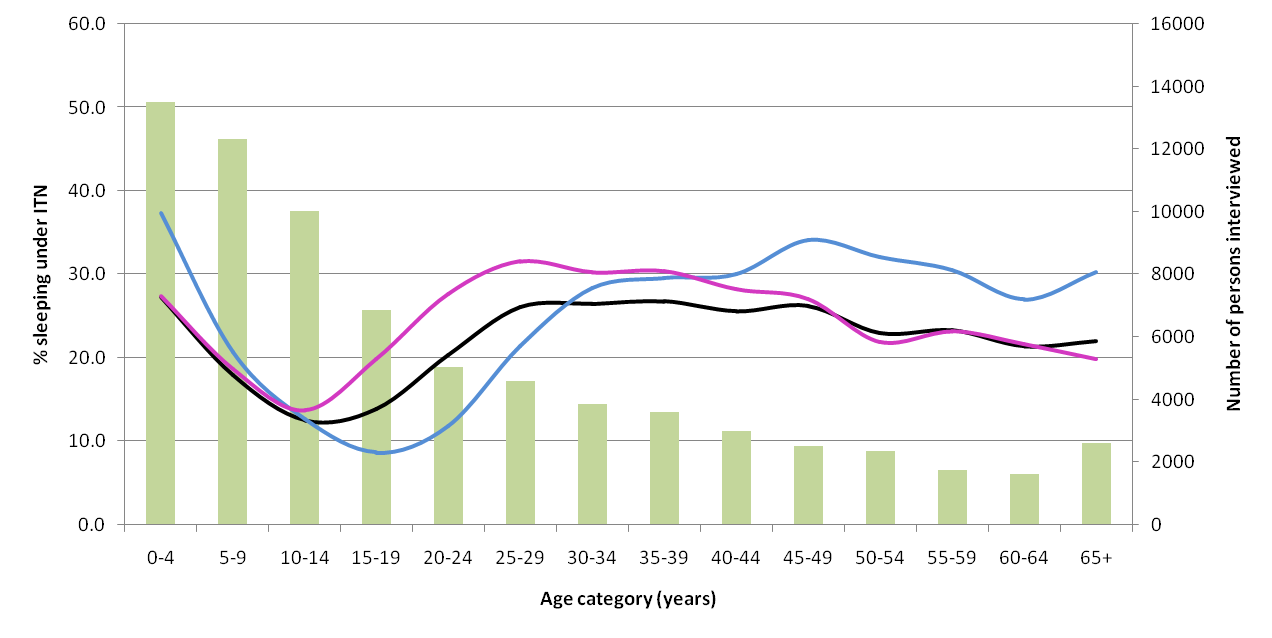


**Group 2 (≥ 10-<20% ITN use among all ages)**

**Benin (DHS 2006)**


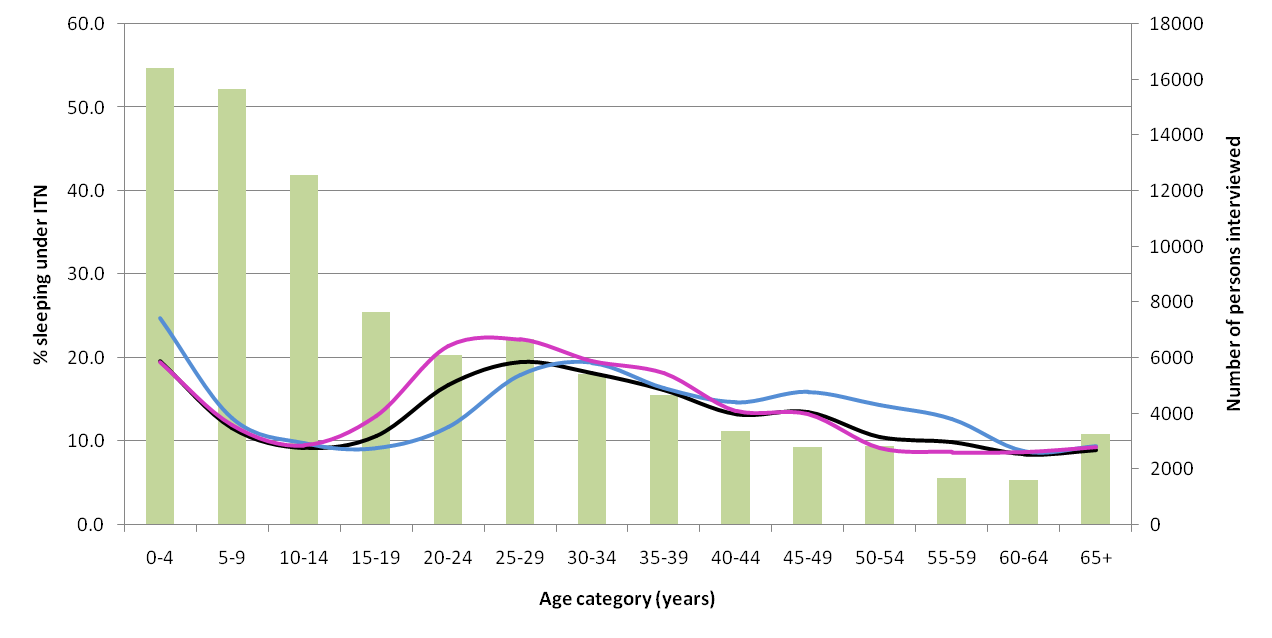


**Senegal (MIS 2006)**


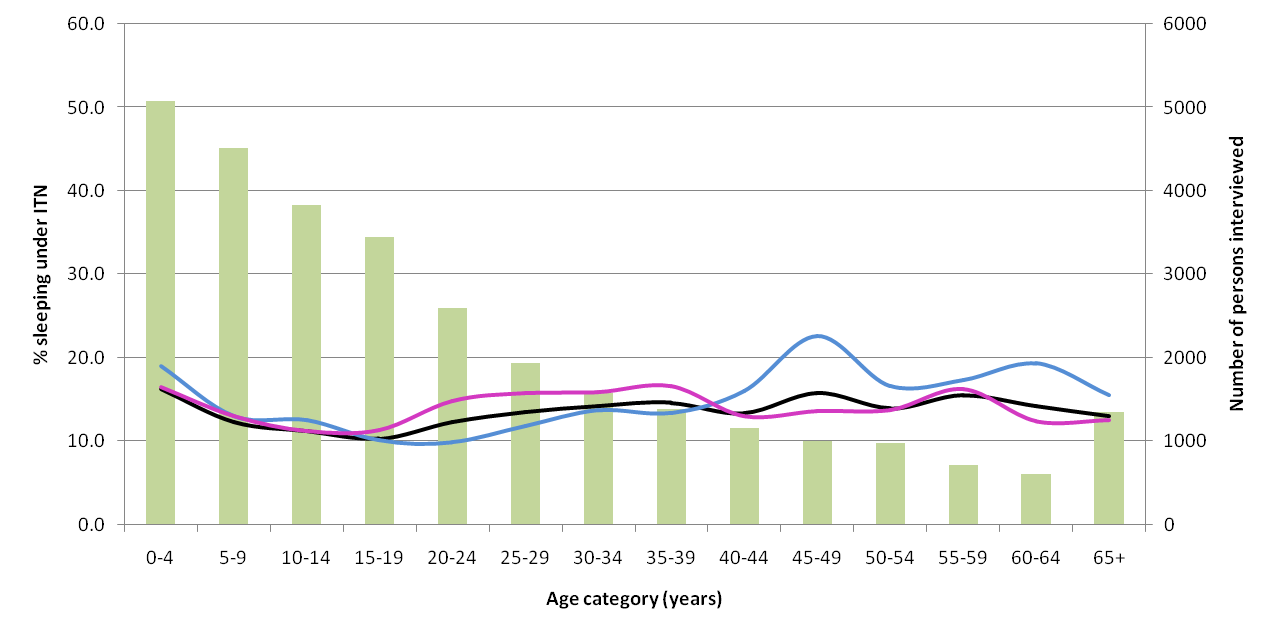


**Angola (MIS 2006-7)**


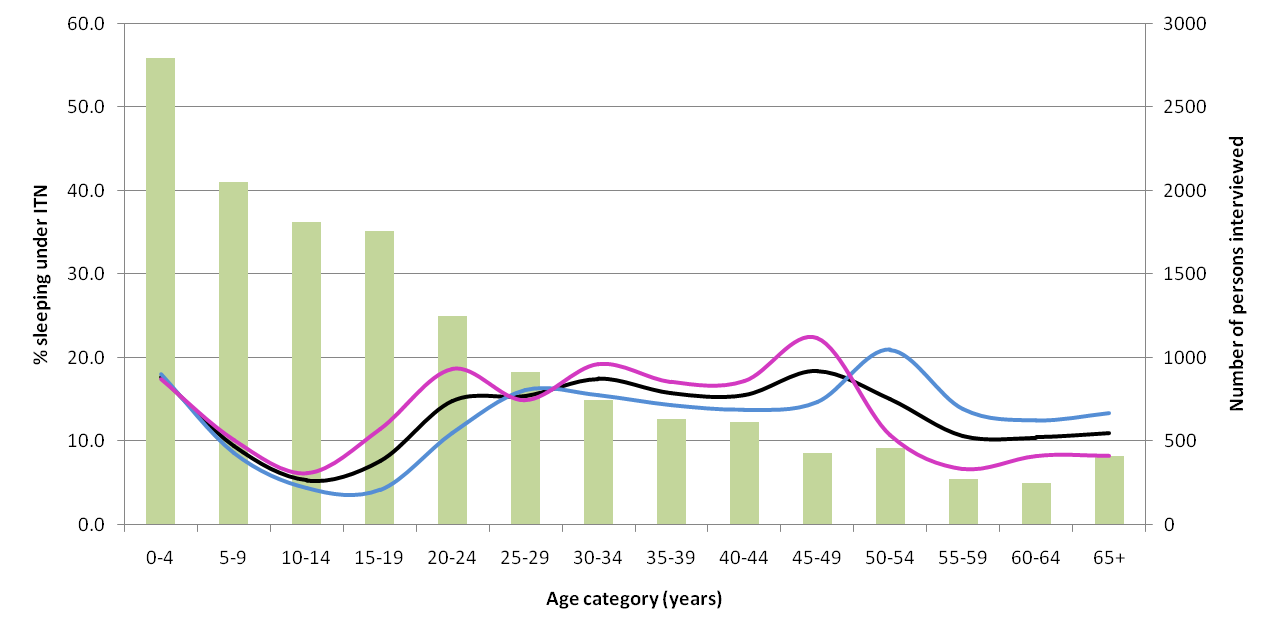


**Djibouti (MIS 2008-9)**


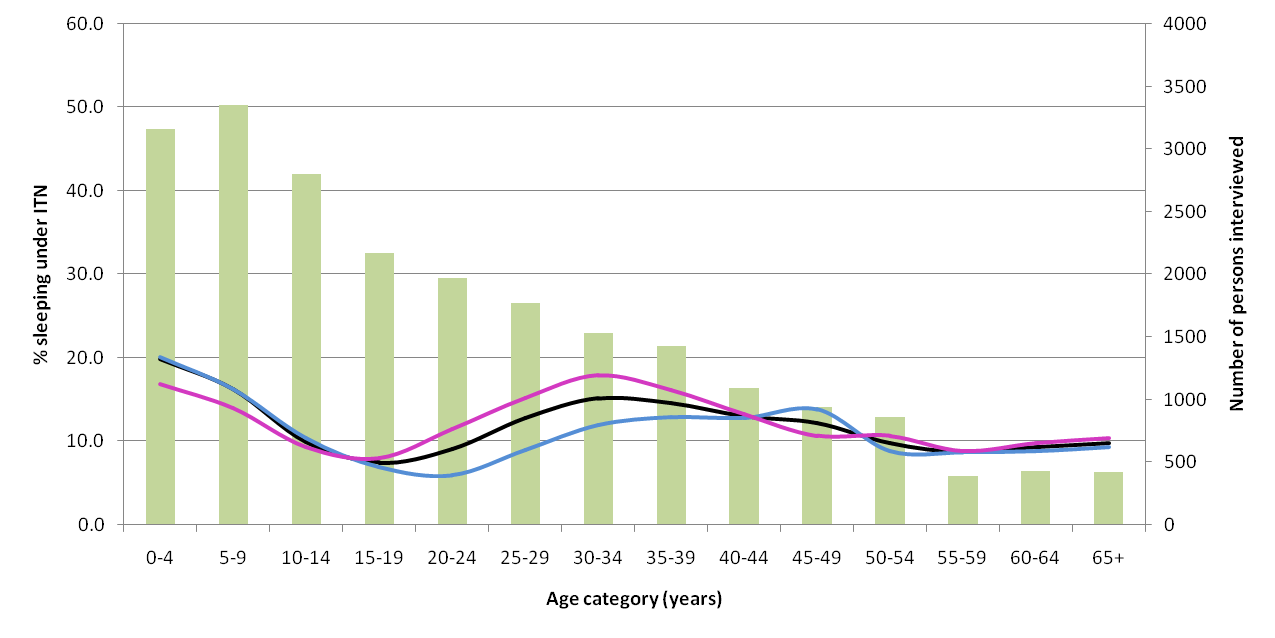


**Sudan (MIS 2005)**

**
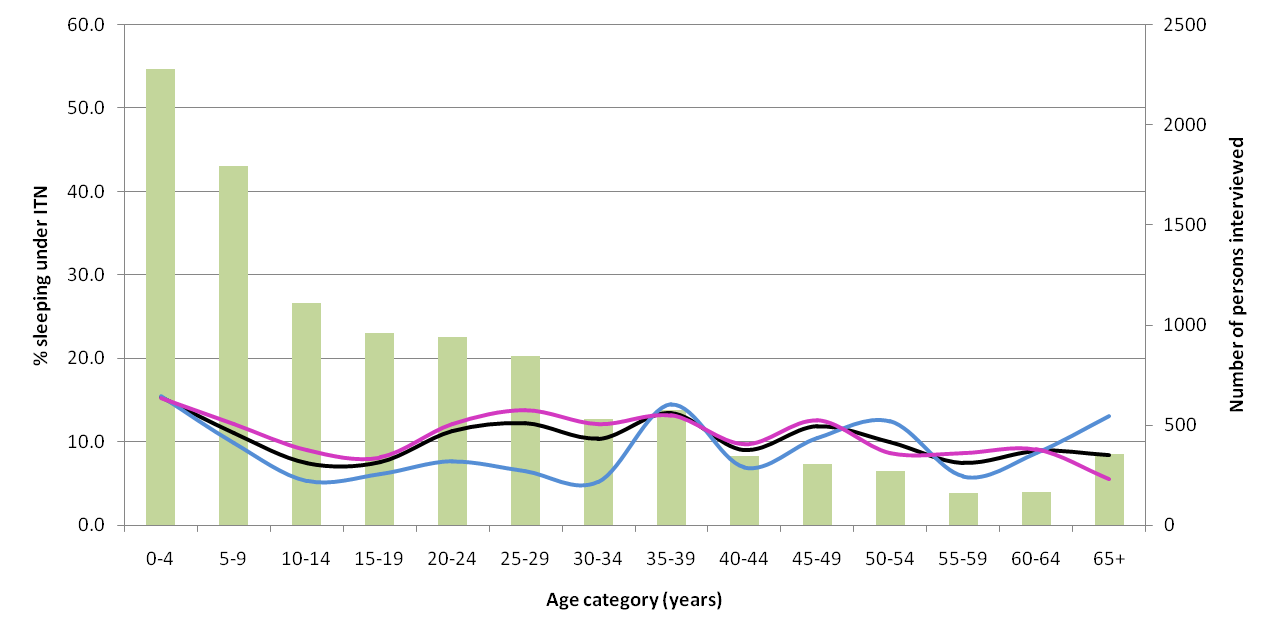
**

**Group 3 (<10% ITN use among all ages)**

**Rwanda (DHS 2005)**

**
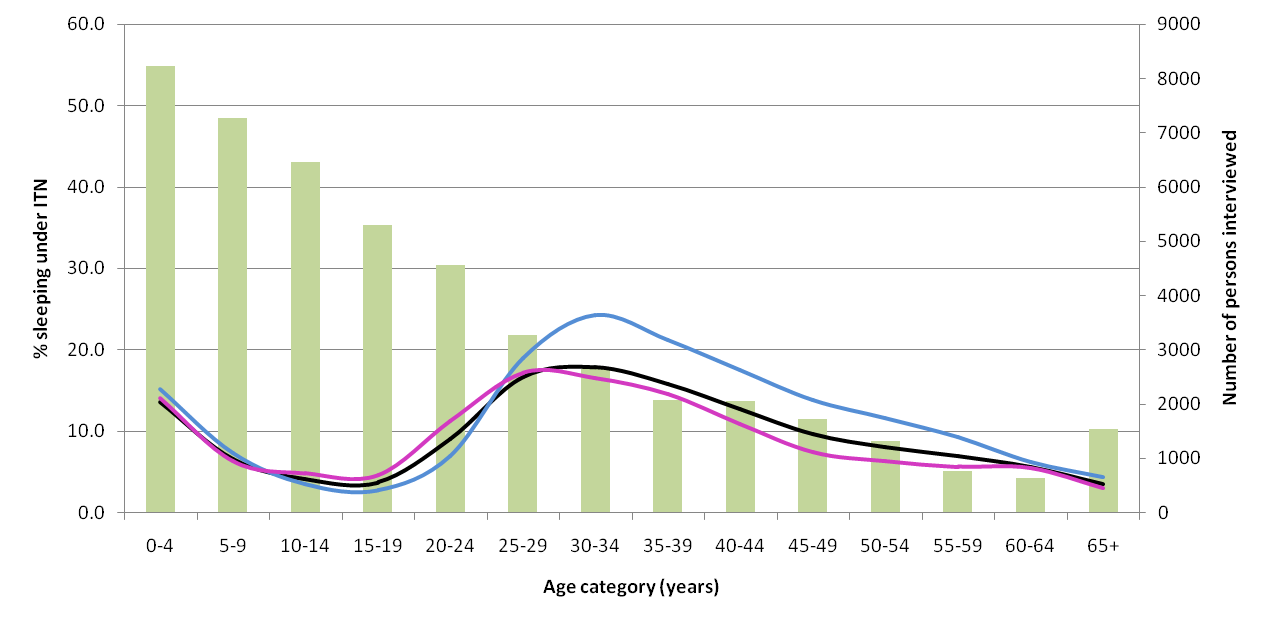
**

**Uganda (DHS 2006)**

**
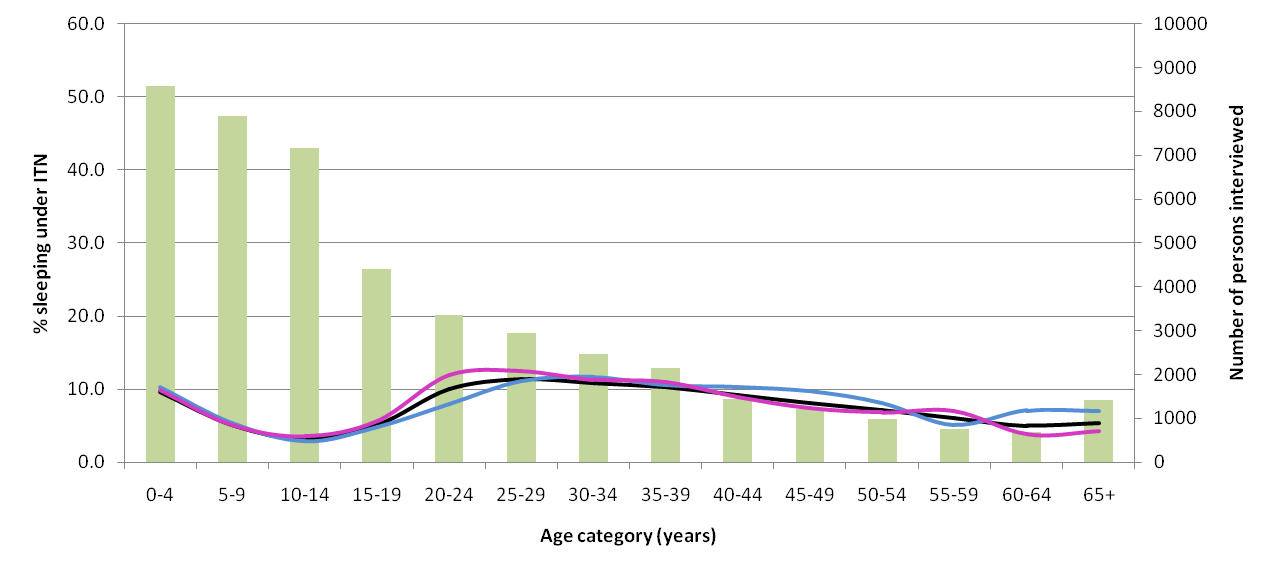
**

**Namibia (DHS 2006-7)**

**
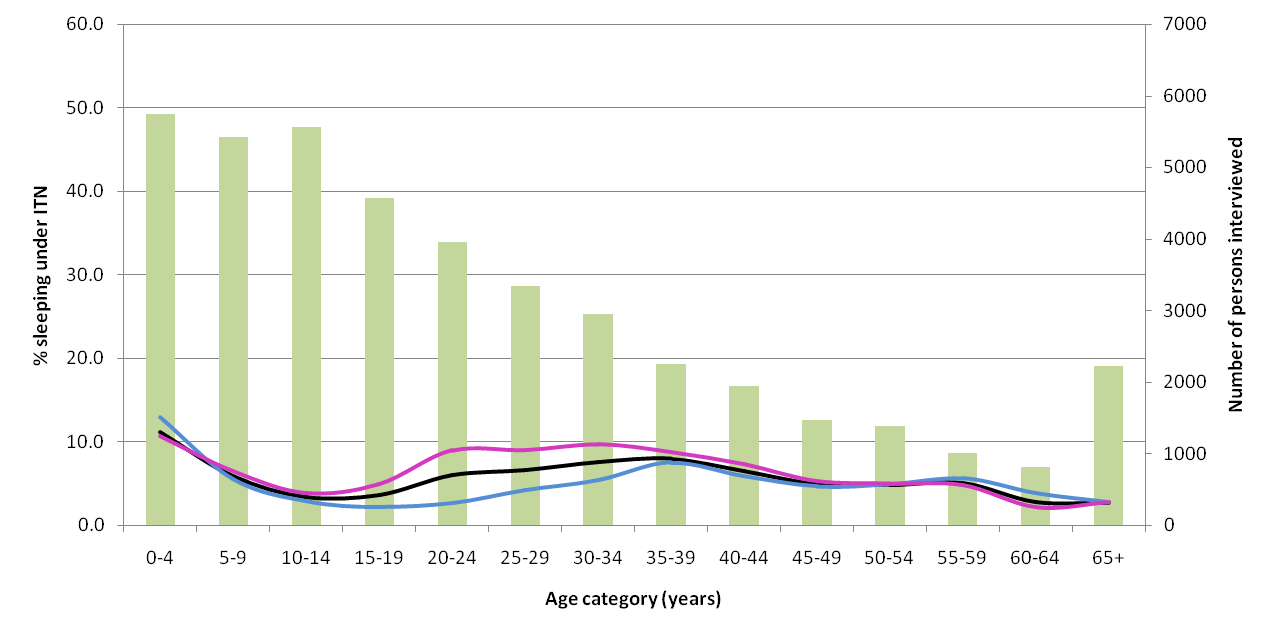
**

**Niger (DHS 2006)**

**
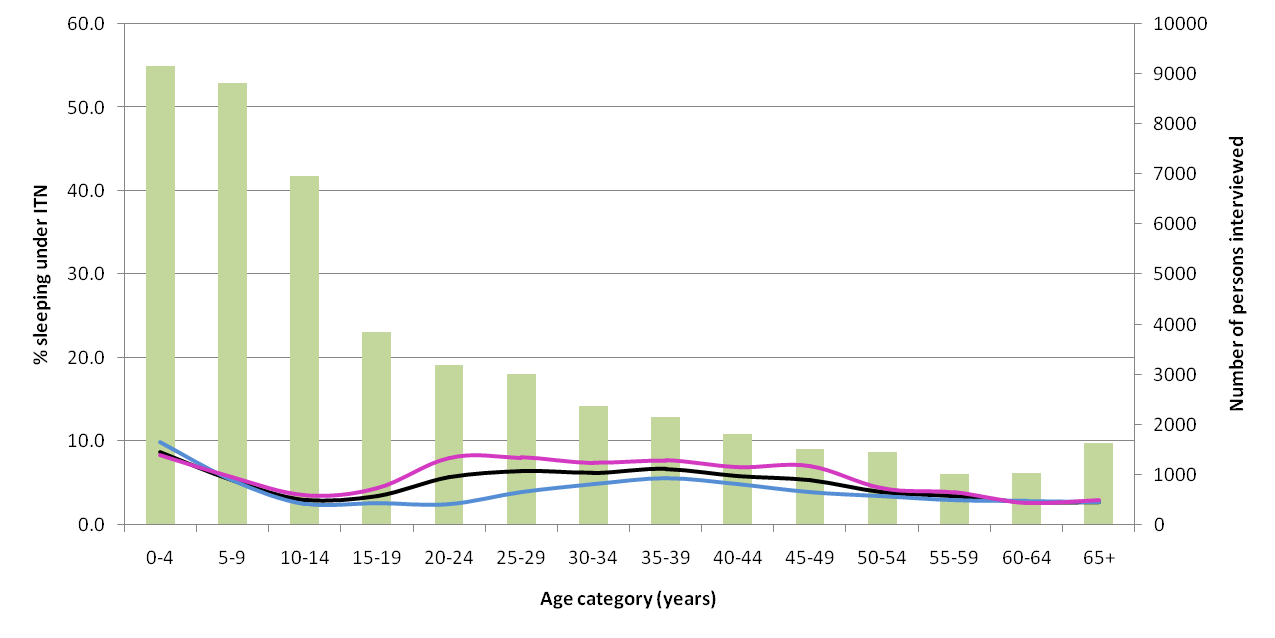
**

**Democratic Republic of Congo (DHS 2007)**

**
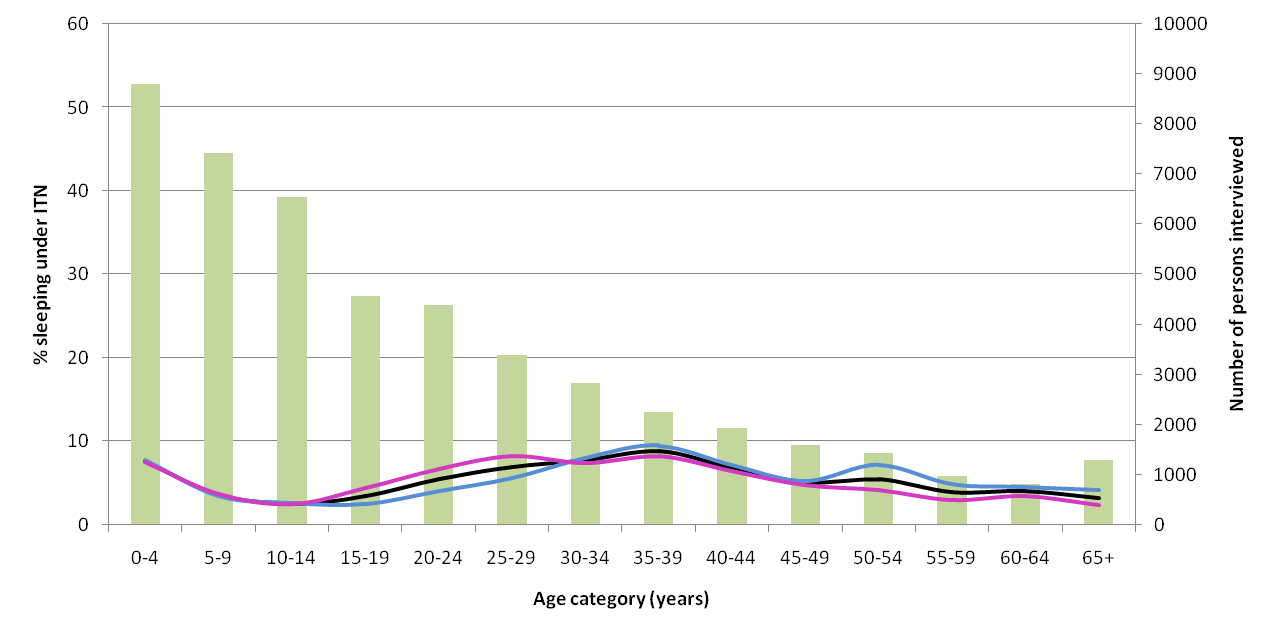
**

**Zimbabwe (DHS 2005-6)**

**
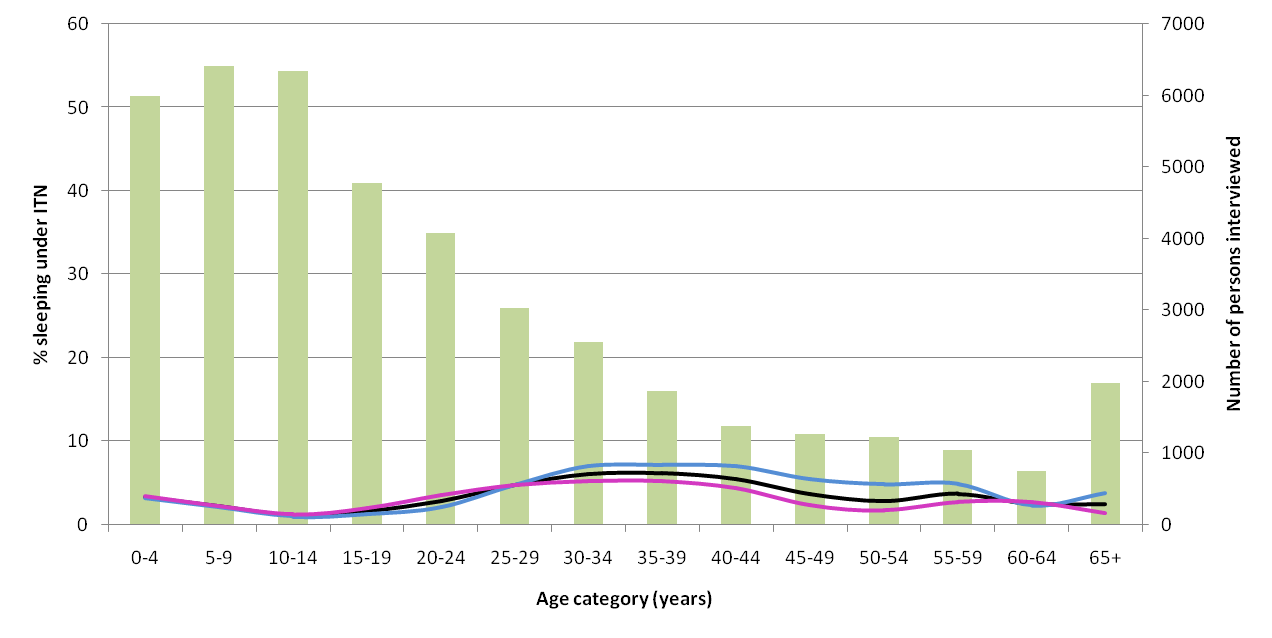
**

**Ethiopia (DHS 2005)**

**
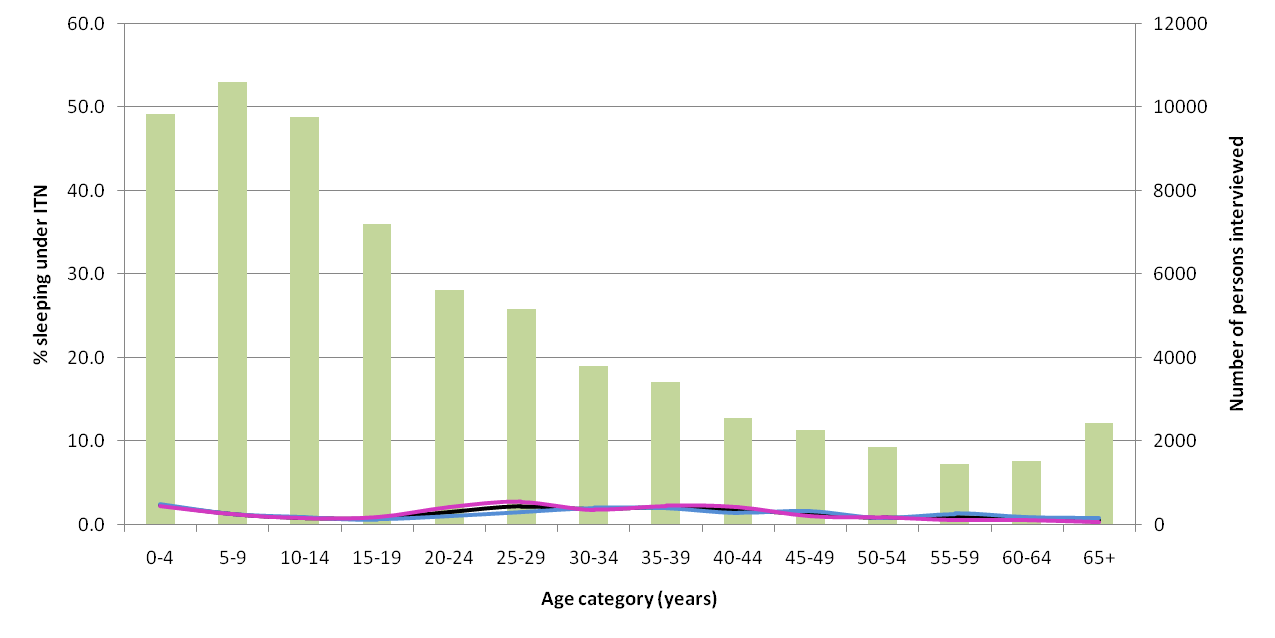
**

**Guinea (DHS 2005)**

**
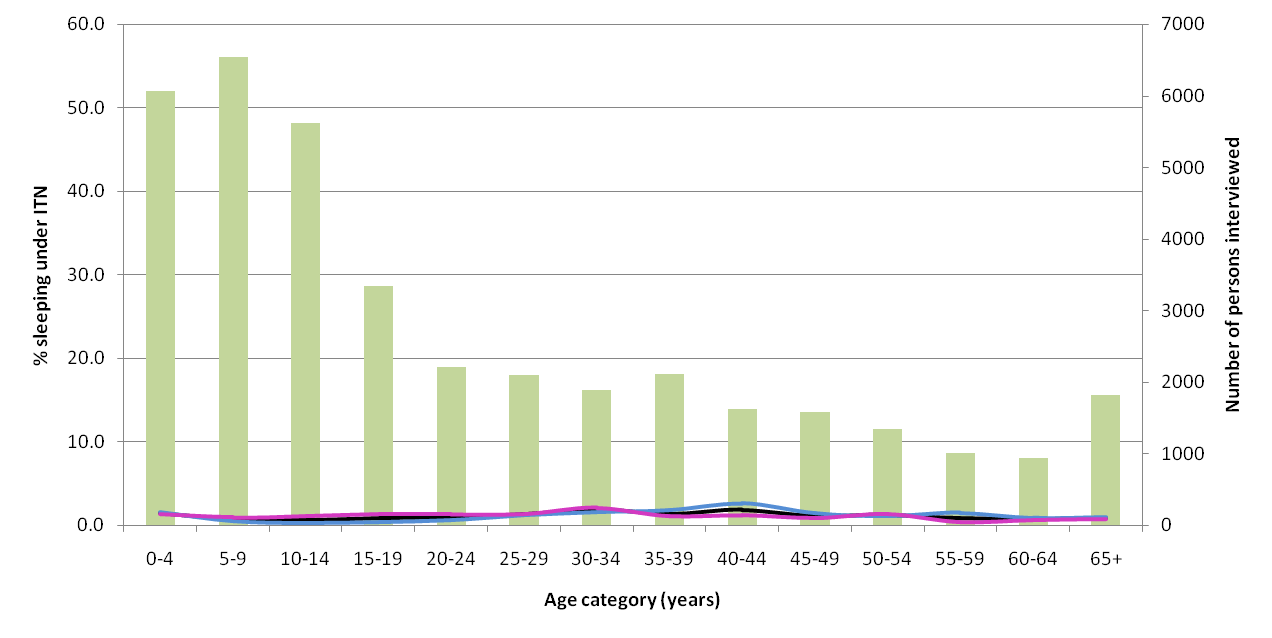
**

**Swaziland (DHS 2006-7)**

**
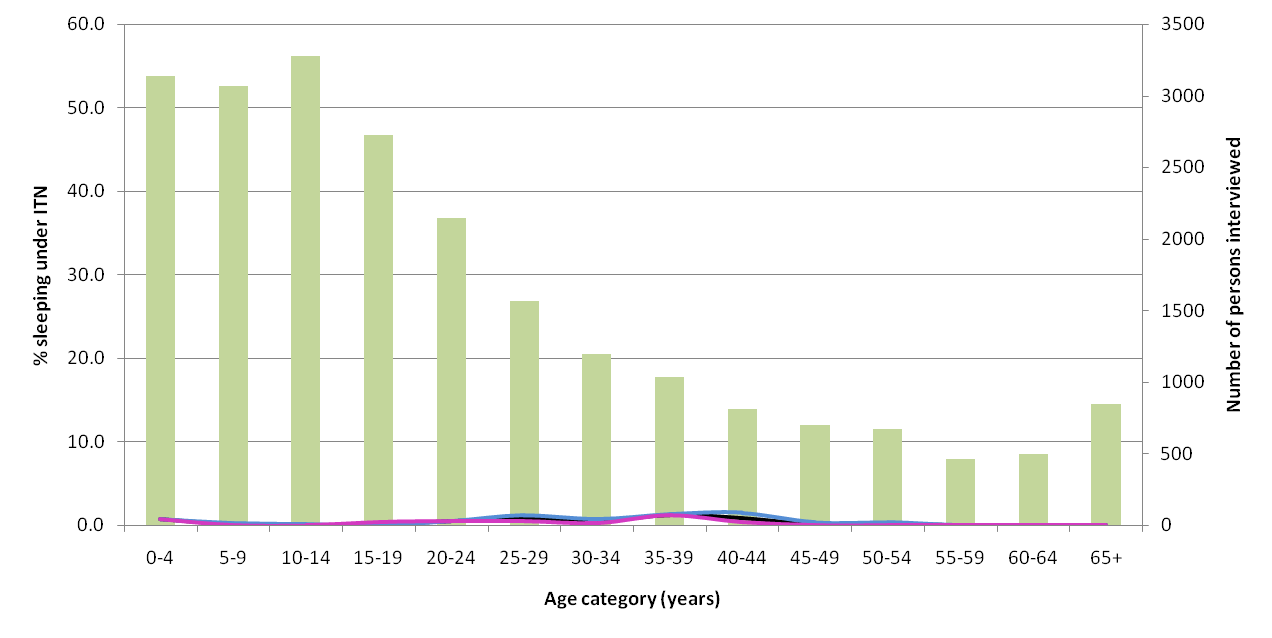
**
